# Insight Into Non-Pathogenic Th17 Cells in Autoimmune Diseases

**DOI:** 10.3389/fimmu.2018.01112

**Published:** 2018-05-28

**Authors:** Xinyu Wu, Jie Tian, Shengjun Wang

**Affiliations:** ^1^Department of Laboratory Medicine, The Affiliated People’s Hospital, Jiangsu University, Zhenjiang, China; ^2^Department of Immunology, Jiangsu Key Laboratory of Laboratory Medicine, School of Medicine, Jiangsu University, Zhenjiang, China

**Keywords:** Th17 cells, IL-10, inflammation, immunosuppression, autoimmune diseases

## Abstract

Th17 cells are generally considered to be positive regulators of immune responses because they produce pro-inflammatory cytokines, including IL-17A, IL-17F, and IL-22. Cytokine production not only promotes accumulation of immune cells, such as macrophages, neutrophils and lymphocytes, at inflammatory sites but can also cause tissue pathologies. Conversely, certain Th17 cells can also negatively regulate immune responses by secreting immunosuppressive factors, such as IL-10; these cells are termed non-pathogenic Th17 cells. In this review, we summarize recent advances in the development and regulatory functions of non-pathogenic Th17 cells in autoimmune diseases.

## Introduction

In the 1990s, the novel cytokine interleukin-17 (IL-17) was identified and cloned from a CD4^+^ T-cell library (1), after which a new type of helper T cells, named Th17 cells, was revealed. Although there are six members of the IL-17 family (A–F), only IL-17A and IL-17F are secreted by Th17 cells (2). Th17 cells also produce other cytokines including IL-22 and IL-21. Retinoid-related orphan receptor gamma t (RoRγt), or its homolog Rorc in humans, is the most specific transcription factor of Th17 cells. IL-17A and/or IL-17F play important roles in many autoimmune diseases, e.g., leading to aggravation of such conditions as experimental autoimmune encephalomyelitis (EAE), psoriasis, rheumatoid arthritis (RA), and Crohn’s disease (3–5).

Due to the pro-inflammatory effects of IL-17A and IL-17F, exploring the origin and function of Th17 cells may be beneficial for inflammatory disease therapy. It is widely acknowledged that Th17 cells can be stimulated by the combination of transforming growth factor-β (TGF-β) and IL-6 or by the IL-23p40 pathway alone. However, a series of experiments have shown that IL-23 does not promote differentiation of naïve T cells into Th17 cells because naïve T cells do not express the IL-23 receptor *in vitro*. Thus, it appears that the main function of IL-23 is to maintain the phenotype of Th17 cells and their survival. Although TGF-β and IL-6 stimulate Th17 cell differentiation, the converse may be promoted depending on the class of TGF-β. TGF-β1/IL-6 induce Th17 cells with IL-10, whereas TGF-β3/IL-6 induce IL-17-producing cells along with conventional Th17 cells (6). Such IL-10-producing Th17 cells do not induce tissue inflammation and in fact inhibit autoimmune inflammation.

Unlike Th1 and Th2 cells, Th17 cells exhibit a high degree of plasticity, trans-differentiating into Th1, regulatory T (Treg), and follicular helper T (Tfh) cells. It has been reported that Th17 cells in the gut of patients with Crohn’s disease or in the joints of those with arthritis co-express IL-17 and interferon-gamma (IFN-γ) (7, 8). This type of Th17/Th1 cell expresses specific transcription factors such as T-bet and RORγt, and high IL-12 or low TGF-γ levels may contribute to differentiation *in vitro*. IL-23 can partly block the function of IL-12 in Th17/Th1 cells, though IL-23 is deemed a promoter in mice (8). In addition, many studies report that IL-17 production by Treg cells is accompanied by increased expression on RORγt and decreased expression of Foxp3 (9, 10). Thus, Th17/Treg cells maintaining Foxp3 exert a suppressive function based on differential expression of CCR6 and IL-1RI (11). Moreover, Th17 cells have been shown to trans-differentiate into Tfh cells in Peyer’s patches, inducing the development of IgA-producing germinal center B cells (12).

However, a new subset of IL-10-producing Th17 cells has been discovered recently. Such IL-10 producing Th17 cells do not induce tissue inflammation and actually inhibit autoimmune inflammation. Here, we review recent literature on both the function and molecular hallmarks of non-pathogenic Th17 cells and the implications of these cells in autoimmune diseases.

## Generation of IL-10^+^ Th17 Cells

Despite the observation mentioned above regarding the possible existence of Th17 cells with regulatory effects, the potential role of Th17 cells with suppressive functions in autoimmune inflammatory diseases and cancer have not been fully elucidated. Hueber et al. (13) reported that using an anti-IL-17A monoclonal antibody to treat inflammatory bowel disease (IBD) patients was inefficient and resulted in disease aggravation, hinting that not all IL-17A-producing Th17 cells induce tissue inflammation and possess pro-inflammatory functions.

Abundant evidence to date indicates the existence of IL-10-producing Th17 cells. However, the generation of IL-10-producing Th17 cells has only been recently appreciated. For example, Newcomb et al. discovered increased levels of IL-10 in mouse Th17 cells polarized in the presence of IL-13, followed by restoration of IL-17 and IL-21 levels after IL-10 neutralization (14). Furthermore, IFN-β, which is considered to be a unique cytokine secreted by type-1 CD4 helper T cells, has been shown to upregulate IL-10 production under Th17-polarizing conditions (15). However, in addition to the effects of cytokines, the experiments conducted by Blasko reveal novel insight into the generation of IL-10^+^ Th17 cells (16). The small molecule G-1 is thought to serve as an agonist of membrane-bound-G-protein-coupled estrogen receptor (GPER), which is expressed on human and mouse immune cells (17). Under Th17-polarizing conditions, G-1 can promote expression of both IL-10 and Foxp3. Therefore, G-1 likely delays the onset of EAE *via* the combined action of IL-10^+^IL-17A^+^ cells and Foxp3^+^ Th17 cells (17, 18). Interestingly, increased IL-10 production in the Th17-polarizing settings can occur under hypoxia without the addition of a specific pathogen (19).

## “Non-Pathogenic” and “Pathogenic” Th17 Cells

Whether naïve T cells become non-pathogenic or pathogenic Th17 cells depends upon the cytokine milieu present during the differentiation process. Treatment of naïve T cells with TGF-β1 and IL-6 might promote the generation of non-pathogenic Th17 cells (20). However, this might be abrogated by exposure to IL-23, resulting in the conversion of non-pathogenic Th17 cells into pathogenic Th17 cells (21). Some studies have also indicated that IL-6/IL-23/IL-1β or other cytokine cocktails without TGF-β may increase expression of the master transcription factor RORα during differentiation (21). Indeed, researchers have found that Th17 cells differentiating under the conditions described above have a function and phenotype similar to that of pathogenic Th17 cells. Cytokines such as granulocyte macrophage-colony-stimulating factor (GM-CSF), prostaglandin E2, and Notch signaling molecule RBPJ are also associated with Th17 pathogenicity (22–24). Studies of the transcriptional signature of non-pathogenic and pathogenic Th17 cells can help in understanding these cell subsets. By comparing gene expression profiles of *in vitro* Th17 cells polarized *via* cytokine combinations that induce non-pathogenic or pathogenic Th17 cells, 233 genes with differential expression between the two Th17 cell subsets were identified. Pathogenic Th17 cells express more effector molecules, including pro-inflammatory cytokines/chemokines such as Cxcl3, Ccl4, Ccl5, IL-3, and IL-22 and transcription factors such as Tbx2 and Stat4, whereas non-pathogenic Th17 cells exhibit upregulation of molecules related to immune suppression, cytokines such as IL-10, and transcription factors such as Ikzf3 (6, 25).

## Mechanisms Involved in Modulating IL-10^+^ Th17 Cell Generation

Although there has been great progress in characterizing the requirements for the generation of non-pathogenic Th17 cells, the mechanism underlying IL-10^+^ Th17 cell generation has not yet been fully elucidated.

Recently, by analyzing and comparing single-cell RNA-Seq profiles of non-pathogenic Th17 cells with those of pathogenic Th17 cells, Wang et al. found that the former cells may predominantly express more CD5-like (CD5L) that Th17 cells converted into a regulatory phenotype (26). CD5L, a member of the scavenger receptor cysteine-rich superfamily, is expressed on macrophages and can act as a receptor of pathogen-associated molecular patterns (PAMPs) (27, 28). Comparing wild-type (WT) non-pathogenic Th17 cells stimulated by TGF-β + IL-6 with CD5L^−/−^ Th17 cells polarized under similar conditions in EAE, upregulation of polyunsaturated fatty acids (PUFAs) and downregulation of saturated fatty acids (SFAs) and monounsaturated fatty acids (MUFAs) was found in WT non-pathogenic Th17 cells (26). Cholesterol metabolites are also an important source of endogenous ligands for RORγt (29). Thus, CD5L may alter the lipid composition of Th17 cells, leading to decreased expression of RORγt ligands in these cells. Moreover, binding by RORγt to the promoter regions of IL-17A, IL-22, and IL-10 has been reported (30); thus, a reduction in RORγt ligand results in reduced transcriptional activity. Increased binding of RORγt to the IL-10 promoter region has been demonstrated in WT Th17 cells treated with PUFAs (26). These data indicate that CD5L promotes the production of IL-10 in Th17 cells by regulating RORγt *via* by fatty acids in cells.

CD39 and CD73 engagement are required for suppression of autoimmune diseases. In a model of experimental colitis in Rag^−/−^ mice, Th17 cells polarized *in vitro* were able to produce IL-10 because they expressed CD39 (31). Furthermore, unconjugated bilirubin (UCB) did not protect mice from experimental colitis if CD39 was deleted *in vivo* (32). CD39 and CD73 are two ectonucleotidases: CD39 is highly expressed on endothelial cells and immune cells in many organs and can hydrolyze ATP to AMP; CD73 is mainly expressed on leukocytes in various tissues and can cleave AMP to adenosine to inhibit ATP-induced cell death (33). In addition, CD39 and CD73 expression on Th17 cells is influenced by factors that induce Th17 differentiation, such as TGF-β and IL-6. Notably, IL-6 can promote STAT3 to upregulate expression of CD39 and CD73, whereas TGF-β through P38 activation can inhibit growth factor independent-1 (Gfi1) expression, leading to increased expression of the ectonucleotidases CD39 and CD73 (34). Thus, CD39^+^CD73^+^Th17 cells may exert their immunosuppressive functions in a STAT3- and p38-dependent manner.

Nonetheless, transcription factors may also be important for the production of IL-10. For instance, c-Maf regulates IL-10 production in T cells in mice. Furthermore, it has been reported that c-Maf regulates IL-10 production during Th17 polarization and that this process relies on STAT3 expression in STAT6- and T-bet-double knockout mice. Loss of STAT3 abolishes TGF-β + IL-6-induced c-Maf expression, and IL-10 does not influence TGF-β-mediated induction of c-Maf and IL-10, suggesting that TGF-β may direct the impact of IL-10 through another pathway (35). Regardless, in Th1 and Th17 cells, c-Maf activation for IL-10 production has been proven to be associated with the MAPK/ERK pathway (36). Thus, c-Maf appears to be a key node that links the STAT3 pathway with the MAPK/EAR pathway, constituting a complex cross-talk network in Th17 cells. By chromatin immunoprecipitation (CHIP) assays, it has been shown that c-Maf physically binds to the promoter region of IL-10 with an Maf recognition element (MARE) motif for transactivation (35, 37).

IKZF3, a member of zinc finger protein family, encodes the transcription factor Aiolos. It is noted that TNF-inhibitor treated human Th17 cells co-express IL-17 and IL-10 while highly expressing IKZF3 (38). Five conserved Aiolos binding sites are present in the human IL-10 promoter, and several Ikaros binding sites (GGGAA) have been detected at the IL-10 locus in mice (39).

In addition, AhR, a member of the basic helix–loop–helix transcription factor family, is expressed by various immune cell types. Once engaged by a ligand, such as TCDD, AhR translocates to the nucleus, and the AhR and ARNT complex binds to the XRES/DRES motif of target gene (40). These findings indicated that AhR along with c-Maf can facilitate IL-27-induced IL-10 production, as AhR physically interacts with c-Maf, the expression of which is limited to Tr1 cells (41). Therefore, the role of AhR in modulating IL-10 production by Th17 cells or other cell types needs to be further assessed.

## Role of IL-10^+^ Th17 Cells in Autoimmune Diseases

**Figure 1:**
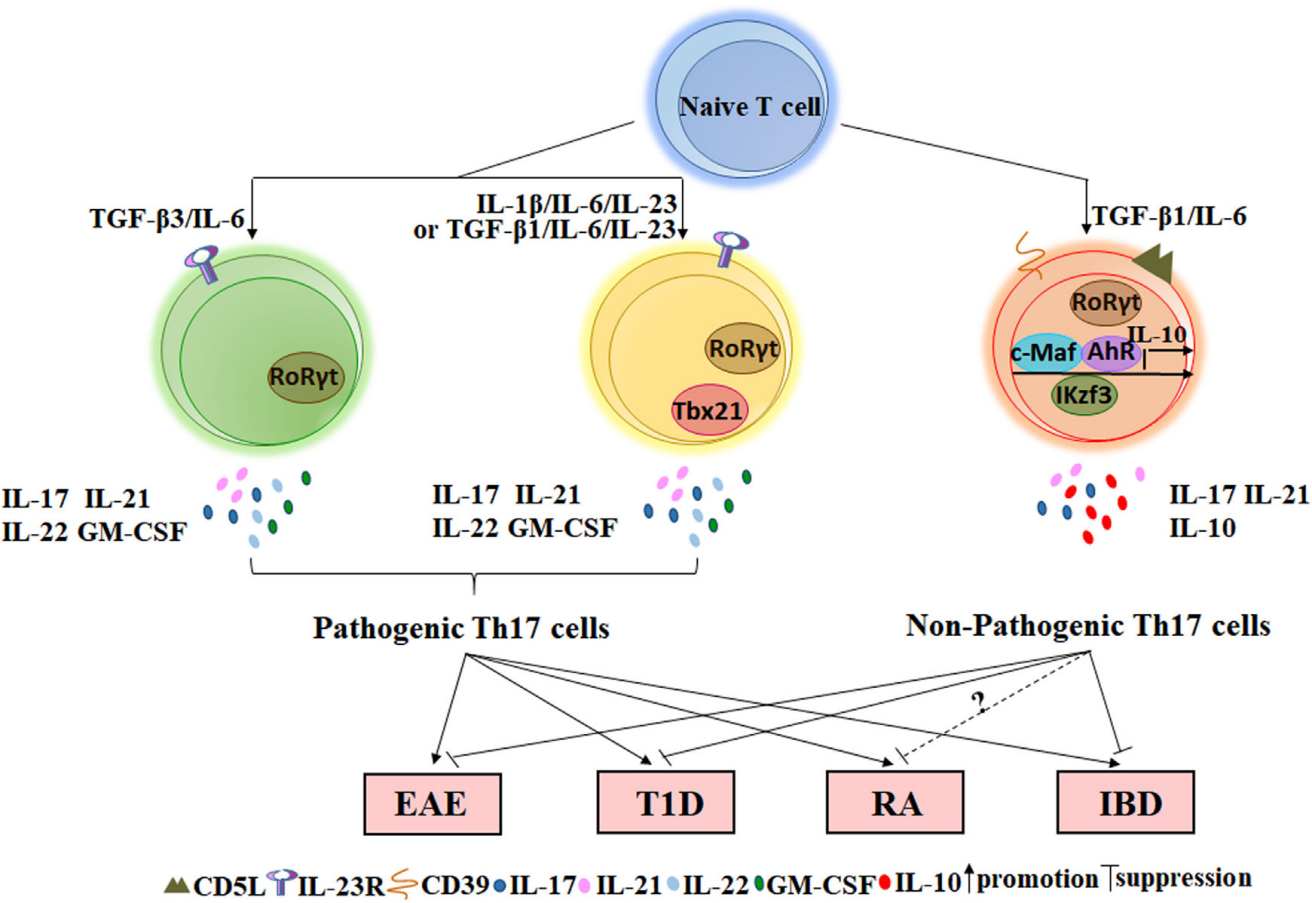
The role of non-pathogenic Th17 and pathogenic Th17 cells on autoimmune diseases. Naïve CD4^+^T cells stimulated with TGF-β3/IL-6, IL-1β/IL-6/IL-23, or TGF-β1/IL-6/IL-23 *in vitro* can secret inflammatory cytokines such as IL-17, IL-21, IL-22, and GM-CSF and express the specific transcription factor RORγt. Such Th17 cells that play important roles in the occurrence of autoimmune diseases are named by pathogenic Th17 cells. However, TGF-β1/IL-6-induced Th17 cells not only express RORγt but also express several IL-10-related transcription factors such as c-MAF, Ikzf3, and AhR. This type of Th17 cell expresses a low level of or lacks IL-23R but expresses a high level of CD5L/AIM and CD39 ectonucleotidases at the membrane. Adoptive transfer of Th17 cells induced by TGF-β1/IL-6 into wild-type mice does not cause EAE or T1D. In addition, a small fraction of Th17 cells in the intestine with suppressive function can also reduce IBD *via* IL-10 and TGF-β. However, the effect of non-pathogenic Th17 cells on RA requires further research in mice. EAE, experimental autoimmune encephalomyelitis; T1D, type 1 diabetes; RA, rheumatoid arthritis; IBD, inflammatory bowel disease.

The regulatory function of IL-10^+^ Th17 cells has been extensively characterized in inflammation, cancer, and autoimmune diseases. Th17 cells are widely thought to play a pathogenic role in the development of autoimmune diseases because they produce pro-inflammatory cytokines that may result in tissue damage. However, these cytokines may also provide protective effects when our bodies detect the presence of foreign pathogens because these molecules contribute to the clearance of bacteria and fungi. For example, IL-17 can indirectly kill *Bordetella pertussis* by activating macrophages, which engulf pathogens (42). Moreover, IL-17AR-knockout mice are more susceptible to *Candida albicans* infection (43). In contrast, cells of the Th17 lineage promote granulocyte induction and neutrophil recruitment to sites of inflammation. IL-23p19 blockade *in vivo* can both decrease IL-17 transcription and translation and also reduce neutrophil trafficking to the lungs of mice in an acute respiratory *Mycoplasma pneumoniae* infection model (44). Thus, current studies on the functional implications of IL-10^+^ Th17 cells in the pathogenesis of autoimmune diseases can direct the development of combined therapies for autoimmune diseases and allow for a better understanding of the functional diversity of Th17 cells. In the next sections, the role of IL-10^+^ Th17 cells in several autoimmune diseases and disease models, such as EAE, type 1 diabetes (T1D), RA, IBD, and psoriasis, will be elaborated (Figure 1).

### Roles of IL-10^+^ Th17 Cell in EAE

Multiple sclerosis is an autoimmune disease that results from immune cells infiltrating the blood–brain barrier (BBB), leading to inflammation, demyelination, and gliosis in the central nervous system. Many studies on myelin-directed autoimmunity are based on the mouse model of EAE induced by immunization with myelin basic protein (45). Increasing evidence suggests that Th17 cells play an important role in the onset of EAE. Komiyama found that the development of EAE was delayed in IL-17^−/–^ knockout mice, as indicated by reduced disease severity scores, fewer marked histological changes, and more rapid recovery from disease (46). Therefore, targeting IL-17 may represent a potentially effective method for treating multiple sclerosis patients.

Compelling evidence is derived from a study in which naïve CD4^+^ T cells from myelin oligodendrocyte peptide [amino acids 35–55 (MOG-35–55)]-specific TCR transgenic mice (2D2) were stimulated with TGF-β1 and IL-6 *in vitro* for 4 days. Induced Th17 cells were then transferred to WT transgenic mice, with no EAE developing; in contrast, Th17 cells polarized in the presence of TGF-β1, IL-6, and IL-23 resulted in in severe EAE symptoms (15). Moreover, if the PLP peptide of amino acids 139–151 [PLP(139–151)] is used to immunize EAE mice, this phenomenon is also observed for TGF-β1 and IL-6. However, the result might be very different when adoptively transferred cells from the draining lymph node are re-stimulated with TGF-β, IL-6, IL-23, and PLP peptide and transferred into naïve recipient mice. Studies have shown that this type of disease progression cannot promote the occurrence of EAE, as TGF-β and IL-6 may not reveal the pro-inflammatory effect of IL-23, which induces EAE (20). Different cell origins, processing methods and proteins used to immunize mice might be responsible for the discrepancy between these two experiments. Furthermore, after co-transferring cells polarized using TGF-β, IL-6 and IL-23 along with an anti-IL-10 monoclonal antibody, the course of EAE is more rapid, and the symptoms are more severe (20). These observations are consistent with the upregulation of IL-10 by TGF-β and IL-6. These data indicate that TGF-β and IL-6 may act both *via* bystander suppression of IL-10 and directly downregulate Th17 cell-related cytokines and chemokines to prevent pathogenic Th17 cells from accumulating at inflammatory sites, leading to reduced leukocyte, mononuclear cell, macrophage, and T-cell infiltration of the central nervous system.

### Roles of IL-10^+^ Th17 Cells in T1D

Type 1 diabetes results from hereditary and environmental factors acting in a genetically susceptible individual to cause organ-specific autoimmune disease through T lymphocyte activation and selective damage of islet cells (47). Currently, it is thought that CD4^+^ or CD8^+^ T cells that are present in islet β cells and secrete corresponding cytokines can drive the development of T1D (48). Non-obese diabetes (NOD) mice are generally used as a spontaneous model to study human T1D. Inhibition of Th17 cells by an IL-17 monoclonal antibody suppresses diabetes development (49), and IL-17-deficient NOD mice display a slower onset of diabetes with decreased islets (50). These data establish the critical role of Th17 cells in the development of diabetes. However, another study involving RNA interference indicated that loss of IL-17 *in vivo* cannot protect transgenic NOD mice from diabetes (51). Moreover, NOD mice injected with complete Freund’s adjuvant (CFA) containing bacillus Calmette–Guérin (BCG) mycobacterium indicated that diabetes may be regulated by Th17 cells. This study showed that CFA injection increased the transcriptional activity of IL-10 and IFN-γ while maintaining expression of IL-17A in pancreatic tissues (52). Therefore, only differences in the diversity of Th17 cells can explain the discordance between these two studies.

Adoptive transfer is the most commonly used technique to examine the influence of a cell on a mouse and its function *in vivo*. One system involves T cells from BDC2.5 T-cell receptor transgenic non-obese diabetic (NOD) mice that are *in vitro*-stimulated with TGF-β/IL-6 or IL-23/IL-6 and PS3 antigen for 5 days, with BDC2.5 spleen cells only stimulated with PS3 antigen used as a control; the frequency and kinetics of disease onset are then monitored after transfer to NOD mice. Using this approach was found that NOD mice receiving Th17 cells (IL-23 + IL-6) showed an earlier occurrence of T1D. Conversely, transferring Th17 cells (TGF-β + IL-6) to mice did not cause T1D. In addition, co-transferring Th17 cells induced under the two types of conditions described delayed the development of disease compared with the transfer of Th17 cells (IL-23 + IL-6) alone (53). This phenomenon is similar to the effect of Th17 cells in EAE that act *via* bystander suppression of IL-10. Therefore, this experiment may indicate that direct interactions occur between non-pathogenic and pathogenic Th17 cells *in vivo*.

### Roles of IL-10^+^ Th17 Cells in RA

Rheumatoid arthritis is a systemic autoimmune disease characterized by inflammation in the synovium. This inflammation is primarily mediated by activated T cells, B cells, and macrophages and leads to chronic synovial damage and cartilage and joint destruction (54). Although Th1 cells were originally thought to be the primary driver of RA development, the role of Th17 cells is receiving increasing attention. To date, studies of IL-10^+^ Th17 cells in RA have been restricted to humans. For example, a co-culture system of monocyte/CD4^+^ T cells from humans cultured with TNF-inhibitor drugs showed conversion of pathogenic Th17 cells into an immunosuppressive phenotype due to increased IL-10^+^ Th17 cells, in turn modulating CD14^+^ monocytes (55). Methotrexate (MTX) and methylprednisolone (MP) treatment of peripheral blood mononuclear cells (PBMCs) from patients with early RA promotes expansion of IL-10^+^Th17 cells (56). The appearance of IL-10^+^ Th17 cells in human peripheral blood indicates that these cells may play an important role in balancing inflammation and the anti-inflammatory response at early stages of the disease, though the detailed functions and roles of these cells in mice should be further explored.

### Roles of IL-10^+^ Th17 Cells in IBD and Psoriasis

Inflammatory bowel disease and psoriasis are characterized by chronic, organotropic inflammation of related tissues. In these two diseases, the immune response is similar and consists of phagocytic cells, dendritic cells (DCs), and natural killer (NK) cells, accompanied by complex antimicrobial peptides and cytokines. The intestine and skin are considered to be a natural immune barrier, and barrier dysfunction occurs in IBD and psoriasis, respectively. Increased permeability of the epithelial barrier in the skin and intestine provide many opportunities for allergens and pathogens to interact with immune cell receptors. IL-17A is expressed by various lineages of innate immune cells, including NK T cells, mast cells, neutrophils, DCs, and γδT cells (57). Abundant IL-17-producing mast cells, neutrophils and γδT cells are also reported in the affected skin of psoriasis patients (58). Unlike murine skin, γδT cells approximately take up 4% of human derma leukocyte without existing in human epidermis (59). These derma γδT cells are a major source of IL-17A production among T cells stimulated by IL-23 or pathogens in the presence of IL-1β, which leads to disease progression (58).

The psoriasis pathological process involves IL-17A-producing γδT cells, whereas commensal bacteria colonization in the gut can promote the generation of Th17 cells in the lamina propria through different mechanisms, and there is an absence of IL-17-producing cells in the lamina propria of germ-free mice (60, 61). It has been demonstrated that IL-17-producing T cells residing in the gut do not induce diseases but rather participate in the clearance of infection and protection of the immune barrier (62). There is also evidence suggesting that neutralization of IL-17 spontaneously causes dextran sulfate sodium-induced colitis and that IL-17R-deficient T cells accelerate gut inflammation in a CD4^+^ T-cell-mediated transfer model of colitis (63, 64). In 2012, it was shown that mice could recover from intestinal inflammation after CD3-specific antibody treatment. In this model, a small fraction of Th17 cells with suppressive function were isolated from the intestine of mice; compared with conventional pathogenic Th17 cells, these rTh17 cells mediated suppressive activities *via* IL-10, TGF-β and CTLA-4. Moreover, it was noted that these Th17 cells potentially migrate from the spleen *via* the CCR6/CCL20 axis or are intestine-resident Th17 cells (65).

In inflammation, Th17 cells typically produce IL-17 and other cytokines that act on IL-17-receptor bearing tissue cells such as keratinocytes, synoviocytes, fibroblasts, and epithelial cells, leading to the pathological damage of related diseases. Therefore, targeting IL-17 is a feasible method for treating autoimmune diseases. Secukinumab is a fully human anti-IL-17A immunoglobulin G 1κ (IgG1κ) monoclonal antibody that selectively binds to and neutralizes IL-17A to alleviate diseases such as psoriasis, psoriatic arthritis and ankylosing spondylitis (66). However, secukinumab has no effect on IBD (13), which may be attributed to the functional transformation of Th17 cells in the background of different diseases, even though Th17 cells are always linked to autoimmunity. In the background of psoriasis, secukinumab only reduces the autoimmune inflammation resulting from IL-17A while leaves other immune functions undisturbed (67). However, in the context of IBD, targeting IL-17A may locally exacerbate homeostasis disorder by overpromoting Th1 cells differentiation (63).

Taken together, non-pathogenic Th17 cells do not necessarily function by secreting IL-10: they can naturally reside in the lamina propria with an immunoregulatory function. Th17 cells can also be induced utilizing adoptive transfer by adding specific cytokines *in vitro* to remedy autoimmune diseases. The density and type of cytokines *in vivo* and the time point for transfer of non-pathogenic Th17 cells may affect the outcome in disease models, which are largely determined by the balance of inflammatory and anti-inflammatory signals. Moreover, the treatment of autoimmune diseases by knocking out IL-17 may generate contradictory consequence, such as T1D or IBD. Commensal microbiota may be responsible for this outcome because bacteria such as segmented filamentous bacteria (SFBs) mediate enhancement of Th17 cell immunity that contributes to the resistance to T1D (68). Considering the complexity and diversity of the intestinal microflora in different autoimmune diseases, an IL-17A monoclonal antibody should be used with caution.

## Conclusion

Th17 cell heterogeneity has been the focus of a significant number of recent research efforts. In this review, we consider the regulatory function of IL-10-producing Th17 cells in autoimmune diseases and the potential factors and signaling mechanisms that can induce IL-10^+^ Th17 cells. As summarized above, IL-10-producing Th17 cells may exert immunosuppressive functions by producing IL-10 to induce bystander suppression but also clear pathogens *via* IL-17A secretion when needed. Nonetheless, IL-10-producing Th17 cells represent a very small fraction of Th17 cells, whether polarized *in vitro* or isolated from tissues *ex vivo*. Although we describe the regulatory effect of these cells in autoimmune diseases, a study by Chang et al. demonstrated that IL-10-producing Th17 cells promote induction of immune tolerance, leading to aggravation of endometriosis (69). Thus, IL-10-producing Th17 cells appear to act as a double-edged sword in the pathogenesis of various diseases, and a number of non-pathogenic Th17 cells can also contribute to the regulation of certain diseases in specific contexts. Despite the ability to distinguish non-pathogenic from pathogenic Th17 cells by gene expression profiling, Th17 cells stimulated by TGF-β and IL-6 can be polarized to non-pathogenic Th17 cells *in vitro*. Moreover, naïve T cells are not only surrounded by TGF-β and IL-6 but are also exposed to pro-inflammatory cytokines such as IL-1β and IL-23, raising the question about how non-pathogenic Th17 cells are naturally derived *in vivo* and whether IL-10-producing Th17 cells represent an intermediate state between Th17 cells and Treg cells (70). Because Treg cells can also express IL-17, they possess some functions that overlap with non-pathogenic Th17 cells (71, 72). After a pathogen enters the body, naïve T cells differentiate into effector T cells and memory T cells after antigen presentation by macrophages or B cells. To date, it is unclear whether non-pathogenic Th17 cells also follow this paradigm to generate memory and effector non-pathogenic Th17 cells. Other unanswered questions include whether pathogenic Th17 cells convert into non-pathogenic Th17 cells during a maturation or differentiation period according to the development of disease and need of the body. Regardless, a growing body of evidence indicates that Th17 cells can trans-differentiate into a Th1 or Th2 cell phenotype in an *in vitro* culture system if suitable cytokines are added (73). Thus, the plasticity of Th17 cells may be dependent on the dominant factor in the surrounding milieu. Considering the complex micro-environment of the human body, advances in mice applied to humans should be considered carefully and warrant more exploration.

## Author Contributions

XW wrote the manuscript and discussed the content with the other coauthors. JT discussed the content with the other coauthors. SW conceived the topic and revised the manuscript.

## Conflict of Interest Statement

The authors declare that the research was conducted in the absence of any commercial or financial relationships that could be construed as a potential conflict of interest.
